# Comparing evolvement of visual field defect in neuromyelitis optica spectrum disorder-optic neuritis and idiopathic optic neuritis: a prospective study

**DOI:** 10.1186/s12886-022-02510-y

**Published:** 2022-08-09

**Authors:** Jiaqi Liang, Yuxin Zhang, Kaiqun Liu, Xiaoyu Xu, Xiujuan Zhao, Wei Qiu, Xinyu Zhang, Hui Yang

**Affiliations:** 1 State Key Laboratory of Ophthalmology, Zhongshan Ophthalmic Center, Sun Yat-Sen University, No.54 Xianlie South Road, Guangzhou, 510060 China; 2 Department of Ophthalmology, Sun Yat-Sen Memorial Hospital, Sun Yat-Sen University, 107 Yanjiang West Road, Guangzhou, 510000 People’s Republic of China; 3 Department of Neurology, The Third Affiliated Hospital of Sun Yat-Sen University, 600 Tianhe Road, Guangzhou, 510630 China

**Keywords:** Optic neuritis, Neuromyelitis optica spectrum disorder related optic neuritis, Idiopathic demyelinating optic neuritis, Visual field

## Abstract

**Purpose:**

To compare the evolvement of visual field (VF) defect in neuromyelitis optica spectrum disorder-optic neuritis (NMOSD-ON) and idiopathic optic neuritis (IDON).

**Methods:**

This prospective study involved 104 optic neuritis (ON) patients followed up for ≥ 6 months (33 patients with NMOSD-ON and 71 patients with IDON). The pattern and recovery pattern of VF defect, mean defect (MD) and pattern standard deviation (PSD) of VF, as well as BCVA at onset, 1 month (1 m), 3 months (3 m), and ≥ 6 months (6 m) after onset were compared between two groups. Analysis of these indicators in first episode patients was also done***.***

**Results:**

Diffuse abnormalities and nerve fiber bundle abnormalities were the two most common patterns in both groups. The percentage of neurologic abnormality of VF defect in NMOSD-ON was higher than that of IDON. Compared with the IDON group, the MD and PSD of NMOSD-ON group were significantly worse at each follow-up. While a positive correlation in BCVA was found between 1 m and ≥ 6 m in the NMOSD-ON group only, a positive correlation was found between 1 m and ≥ 6 m in MD and PSD of both groups. A positive correlation was found between 3 m and ≥ 6 m in MD, PSD and BCVA of both groups. The quadrant recovery pattern was the most common pattern in both groups (57.1% in NMOSD-ON and 57.4% in IDON). The analysis of the first episode subgroup further confirmed the observation above.

**Conclusions:**

The NMOSD-ON patients tended to suffer more severe VF damage, VF irregularity and worse prognosis than that of IDON patients. Diffuse abnormalities and nerve fiber bundle abnormalities were the two most common types in both groups, while neurologic abnormality more common in NMOSD-ON and central scotoma more common in IDON. The visual functions of 1 m in NMOSD-ON and 3 m in IDON were related to its prognosis.

## Introduction

Optic neuritis (ON) is an inflammatory disease involving the optic nerve and causing acute vision loss [1]. ON might be the first manifestation of multiple sclerosis (MS), neuromyelitis optica (NMO) or myelin oligodendrocyte glycoprotein associated disorders (MOGAD) [2–4]. The International Panel for NMO Diagnosis achieved consensus that ON with aquaporin-4 antibody (AQP4-Ab) seropositivity can be diagnosed as neuromyelitis optica spectrum disorder related ON (NMOSD-ON) in 2015 [3]. NMOSD-ON is distinguished as a specific disease from other types of ON based on AQP4-Ab test [5, 6]. In terms of pathogenesis, NMOSD is primarily an astrocyte disease with demyelination as a secondary involvement, while idiopathic demyelinating optic neuritis (IDON), which might evolve to MS related ON (MS-ON), is a primary demyelinating disease [6, 7]. Compared with IDON, NMOSD-ON patients have more female preponderance, more bilateral involvement, worse visual prognosis, and higher relapse rate [7, 8].

The visual field (VF) test helps to locate and quantify the degree of impairment in the affected visual pathway. As an astrocyte disease, NMOSD-ON is different from other types of ON, resulting in different clinical characteristics. As to the severity of VF damage, it is well documented that NMOSD were severer than that of IDON [9]. However, the VF evolvement in NMOSD-ON is not very well characterized. The recent retrospective studies [10–12] showed NMOSD-ON patients had a higher incidence of non-central scotoma than that in IDON, and altitudinal hemianopia may be a characteristic pattern in NMOSD-ON. But no long-term prospective studies about the VF defect of NMOSD-ON in the acute phase were reported.

Therefore, the purpose of this study was to compare the evolvement of VF defect by analyzing the pattern, severity and recovery pattern in patients with NMOSD-ON and IDON by longitudinal and regular follow-up until at least 6 months after onset. We also evaluated possible correlations of MD, PSD of VF and BCVA at different follow-up in both groups of patients.

## Patients and methods

A total of 104 patients with a clinically definite diagnosis of ON in Zhongshan Ophthalmic Center, Sun Yat-sen University from February 2012 to July 2017, were enrolled. Patients were within 14 days after onset, aged from 18 to 65 years old, with a at least 6-month follow-up. This study adhered to the tenets of the Declaration of Helsinki and was approved by the Clinical Research Ethics Board at the Zhongshan Ophthalmic Center of Sun Yat-sen University (2014meky049) and registered on ClinicalTrial.gov (NCT02886377). Informed consent was obtained from all patients included in the study.

Patients participating in this study met the inclusion criteria in accordance with the optic neuritis treatment trial (ONTT) [13]. Patients of acute optic neuropathy with any clinical or laboratory evidence of compressive, ischemic, toxic, hereditary, metabolic, or other ocular or nerve system diseases were excluded. The ON patients with systemic diseases were also excluded, as previously described [14]. The patients were divided into NMOSD-ON group (*n* = 33) and IDON group (*n* = 71). The patients in the NMOSD-ON group met the established diagnostic criteria for NMOSD published by Wingerchuk et al. [3]. The patients in the IDON group fulfilled MS McDonald’s criteria [15], including those with typical acute demyelinating ON and AQP4-Ab seronegative. The patients with AQP4-ab negative fulfilled NMOSD criteria were also excluded. All patients were treated with corticosteroids and neurotrophic drugs, such as methylcobalamin, citicoline and Vitamin B Complex Tablets.

At first visit, all patients with complete medical histories underwent routine neurological examinations, magnetic resonance imaging (MRI) for brain and eye, and ophthalmological examinations, including best corrected visual acuity (BCVA, converted to the logarithm of the minimal angle of resolution (log MAR) for statistical analysis), intraocular pressure, slit lamp and fundus examination, VF (STATPAC, Allergan Humphrey, San Leandro, Calif), VEP (Espion, Diagnosys, America), optical coherence tomography (OCT, Heidelberg Engineering, Heidelberg, Germany). Laboratory testing, including blood routine, infection-related antibodies detection, extractable nuclear antigen antibodies (SSA/SSB), antinuclear antibody (ANA), rheumatoid factor (RF), anti-double standard deoxyribonucleic acid (anti-ds DNA), anti-cardiolipin antibodies (ACLs), and AQP4-Ab, were also tested [14]. All serum AQP4-Ab was tested by cell-based analysis using human AQP4-transfected cells from a commercial BIOCHIP kit (Euroimmun, Germany) [16]. Each patient was followed at 1 month after onset (1 m), 3 months after onset (3 m), 6–12 months after onset (≥ 6 m). And a thorough eye exam was conducted at each follow-up, including BCVA, intraocular pressure, slit lamp and fundus examination, VF, VEP, OCT.

The automated static 30–2 VF test program: a full-threshold test strategy, a 31.5-apostilb background, and a III size target for the test and blind spot check with foveal threshold and fluctuation tests, was applied. The build in software of Humphrey Field Analyzer (STATPAC, Allergan Humphrey, San Leandro, Calif) was used. The VF result was considered reliable if the fixation losses were below 20%, and false positive and false-negative errors were below 33%. VF tests were not performed on the affected eyes of the patients whose BCVA below 1.0 (log MAR). These patients were considered to the classification in failed VF exam. The indexes of mean deviation (MD) and pattern standard deviation (PSD) were measured to quantify.

About pattern classification, the VF abnormalities were ranked in 21 categories according to Keltner’s improvement based on ONTT [17]. These abnormal VF classification categories were divided into 7 broad groups: diffuse, nerve fiber bundle, central, neurologic abnormalities, artifactual abnormalities, missing data and eyes failed VF exam. The multiple foci, widespread and total loss of vision were included in the diffuse abnormalities. The temporal wedge, enlarged blind spot, nasal step, paracentral, partial arcuate, arcuate and altitudinal were included in the nerve fiber bundle abnormalities. The centrocecal and central abnormalities were included in central abnormalities. The vertical step, quadrant, partial hemianopia, hemianopia and three quadrants were included in the neurologic abnormalities. Artifactual abnormalities were divided into peripheral rim, partial peripheral rim, superior depression and inferior depression. The patients with poor BCVA worse than 1.0 Log Mar were added into the subclassification (Eyes failed VF exam). The patients lost to follow up were included in the missing data group. And for recovery pattern classification, the central recovery was regarded as starting from the central 10° area and gradually spread out. The quadrant recovery was considered to start from one quadrant and then to the neighboring quadrant. And the diffuse recovery was thought to start evenly from the whole area. All the VF patterns were analyzed in a masked method. Three qualified readers (H. Y., J. L., and Y. Z.) reviewed the VF results in a blinded fashion, and then the VF results for each patient were presented in order so that the recovery pattern could be assessed. The final classification was determined by unanimous agreement among all 3 readers.

The data was computerized and analyzed in a strict manner using SPSS 22.0 (SPSS Inc., Chicago, IL, USA). All quantitative data were expressed as mean ± standard deviation. The following tests were used for statistical analysis between two groups: Chi-square test and Fisher exact test for frequency comparisons, Student’s t-test for comparing means, and Pearson linear correlation analysis for correlation detection of MD, PSD of VF and BCVA followed normal distribution. The differences of MD, PSD of VF and BCVA among follow-up time points within each group were compared with analysis of variance. The value of *p* < 0.05 was considered statistically significant.

## Results

### Demographic features (Table 1)

**Table 1:** Demographic features of NMOSD-ON and IDON

**Baseline characteristics**	**NMOSD-ON (** ***n*** ** = 33)**	**IDON (** ***n*** ** = 71)**	***P***
**Age at onset (years)**	32.09 ± 15.08	31.79 ± 17.19	0.699
**Sex ratio F/M (%F)**	27/6 (81.8%)	36/35 (50.7%)	0.001*
**Affected eye**			0.682
**Monocular**	20 (60.6%)	41 (56.3%)	
**Binocular**	13 (39.4%)	30 (43.7%)	
**Previous attack times**			0.090
**0**	16 (48.5%)	52 (73.2%)	0.014*
**1**	5 (15.2%)	12 (16.9%)	0.822
** ≥ 2**	12 (36.3%)	7 (9.9%)	0.001*

*NMOSD-ON* Neuromyelitis optica spectrum disorder- optic neuritis, *IDON* Idiopathic optic neuritis, *P P* value

^*^P < 0.05

The demographic data of the study cohort is summarized in Table 1. A significant female predominance was found in the NMOSD-ON group (81.8% versus 50.7%, *p* = 0.001), which was consistent with other reports [18]. The percentage of relapse ON was significantly higher in the NMOSD-ON group (51.5% in the NMOSD group versus 26.8% in IDON group, *p* = 0.014). While the percentage of first episode ON (48.5% in NMOSD-ON group versus 73.2% in IDON group, *p* = 0.014) was significantly higher in the IDON group. No statistical significance difference was found between the two groups regarding the age at onset and the unilateral or bilateral affected eyes.

### Pattern of VF defect (Fig. 1)

**Fig. 1:**
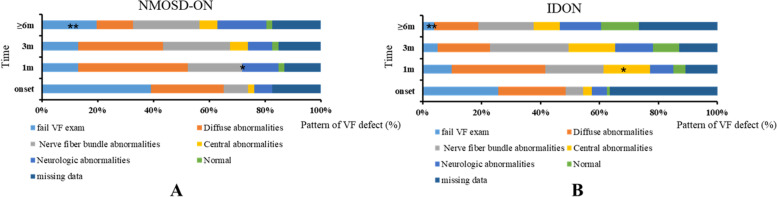
Pattern spectrum of VF defect in NMOSD-ON and IDON. **A** The distribution of patterns of VF defect in NMOSD-ON group at each follow-up. **B** The distribution of patterns of VF defect in IDON group at each follow-up. At 1 m, no central abnormality presented in NMOSD group while in IDON group 15.8% had this pattern (*p* = 0.01 with *). At ≥ 6 m, 4% in IDON group versus 19.6% in NMOSD-ON group failed the VF exam (*p* = 0.005 with **). No significant difference was found between the two groups at other time points (*p* > 0.05). NMOSD-ON: neuromyelitis optica spectrum disorder- optic neuritis, IDON: idiopathic optic neuritis, *P*: *P* value, *: *P* < 0.05, **: *P* < 0.01, onset: at onset of optic neuritis, 1 m: at 1 month after onset, 3 m: at 3 months after onset, ≥ 6 m: at 6–12 months after onset

The patterns of VF defect in two groups at each follow-up are shown in Fig. 1. At onset, the proportion of eyes that failed the VF exam due to poor BCVA in IDON and that in the NMOSD-ON group was 25.7% and 39.1%, respectively. No significant statistical difference was found (*p* = 0.10). Among those who had VF results, the diffuse abnormality was the most common pattern (26.1% in NMOSD group and 22.8% in IDON group). The percentage of nerve fiber bundle abnormality was 8.7% in the NMOSD group and 6% in the IDON group. The percentage of central abnormality was 2.2% in the NMOSD group and 3% in the IDON group. But no significant difference of these patterns was found between the two groups (*p* > 0.05).

At 1 m, the major VF pattern was still diffuse abnormality in both groups (39.2% in NMOSD-ON group versus 31.7% in IDON group, *p* = 0.377). The proportion of eyes that failed the VF exam was reduced to 13% in the NMOSD-ON group and 9.9% in the IDON group (*p* = 0.571). None of the patients in the NMOSD group presented central abnormality. The proportion of central abnormality was 15.8% in IDON group (*p* = 0.003). The percentage of nerve fiber bundle abnormality was 19.5% in the NMOSD group and 19.8% in the IDON group. No statistically significant difference was found between the two group in diffuse abnormality and nerve fiber bundle abnormality at 1 m (*p* > 0.05).

At 3 m, the diffuse abnormality was the most common pattern in the NMOSD group (30.4% versus 17.8% in IDON group), while in the IDON group the nerve fiber bundle abnormality was the most common pattern (26.7% in IDON group versus 23.9% in NMOSD-ON group). The proportion of central abnormality was 6.5% in the NMOSD group and 15.8% in the IDON group. The percent of eyes failed the VF exam was further reduced to 13% in the NMOSD-ON group and 5% in the IDON group. But still no significant difference in the proportion of these patterns was found between the two groups (*p* > 0.05).

At ≥ 6 m, the proportion of eyes that failed the VF exam was further reduced, especially in the IDON group (4% in IDON group versus 19.6% in NMOSD-ON group, *p* = 0.005). Among those who had VF results, nerve fiber bundle abnormality was the most common pattern (21.7% in NMOSD group and 18.9% in IDON group). 15.2% of patients in the NMOSD group and 14.9% of patients in the IDON group presented as diffuse abnormality. The percent of central abnormality was 6.5% in the NMOSD group and 8.9% in the IDON group. But no significant difference in the proportion of these patterns was found between the two groups (*p* > 0.05).

### Evolvement of MD, PSD of VF and BCVA (Fig. 2)

**Fig. 2:**
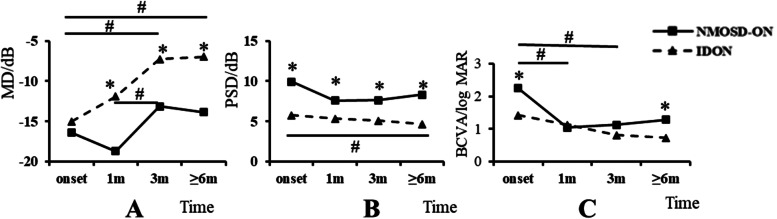
Evolvement of MD, PSD of VF and BCVA in NMOSD-ON and IDON. **A** The evolvement of MD in NMOSD-ON and IDON. The MD in NMOSD-ON group was significantly less than IDON group (*). Both groups began to show significant improvement after 3 m (#). **B** The evolvement of PSD in NMOSD-ON and IDON. The PSD in NMOSD-ON group was significantly higher than IDON group (*). No significant evolvement was found within NMOSD-ON group. In IDON group, a significant difference to onset was found at ≥ 6 m (#). **C** The evolvement of BCVA in NMOSD-ON and IDON. The BCVA in the IDON group was significantly better than NMOSD-ON group at onset and ≥ 6 m (*). In NMOSD-ON group, significant improvement was found at 1 m and 3 m (#), while in IDON group, no significant improvement was found. NMOSD-ON: neuromyelitis optica spectrum disorder- optic neuritis, IDON: idiopathic optic neuritis, MD: mean defect, PSD: pattern standard deviation, BCVA: best corrected visual acuity (log MAR), *P*: *P* value, *: *P* < 0.05, the significant difference between two groups, #: *P* < 0.05, the significant difference between each follow-up, onset: at onset of optic neuritis, 1 m: at 1 month after onset, 3 m: at 3 months after onset, ≥ 6 m: at 6–12 months after onset

To dynamically visualize the changes in the severity of ON in the two groups, we monitored the changes of MD and PSD of VF and BCVA at onset and each follow-up showed in Fig. 2. The average MD of the NMOSD-ON group was similar to the IDON group at onset (-15 ± 10.86 dB versus -16.4 ± 7.70 dB, *p* = 0.680). However, the MD in the NMOSD-ON group was found significantly less than that in the IDON group throughout the follow-ups of ON attack (-18.7 ± 10.80 dB versus -11.9 ± 12.78 dB at 1 m, *p* = 0.020; -13.13 ± 9.42 dB versus -7.24 ± 9.42 dB at 3 m, *p* = 0.002; -13.86 ± 8.92 dB versus -6.98 ± 8.47 dB at ≥ 6 m, *p* = 0.001). As to MD in the NMOSD-ON group, it was significantly better at 3 m than that at 1 m (*p* = 0.033). As to MD in IDON group, it was not found significantly different to onset until 3 m (between onset and 3 m, *p* = 0.039; between onset and ≥ 6 m (as final MD), *p* < 0.001).

The PSD in NMOSD-ON group was significantly higher than that in the IDON group at onset and each follow-up of ON attack (9.93 ± 3.63 dB versus 5.75 ± 3.63 dB at onset, *p* = 0.001; 7.6 ± 4.41 dB versus 5.31 ± 3.19 dB at 1 m, *p* = 0.010; 7.62 ± 4.37 dB versus 5.07 ± 3.49 dB at 3 m, *p* = 0.003; 8.29 ± 4.46 dB versus 4.66 ± 3.30 dB at ≥ 6 m (as final PSD), *p* < 0.001). As to PSD in NMOSD-ON group, no significant difference was found between each follow-up. As to PSD in IDON group, no significant difference to onset was found until ≥ 6 m (*p* = 0.026).

At onset and ≥ 6 m, the BCVA in the IDON group was significantly better than that in the NMOSD-ON group (log MAR, 1.42 ± 1.80 versus 2.25 ± 2.01, *p* = 0.022; 0.73 ± 0.85 versus 1.28 ± 1.75, *p* = 0.026).As to BCVA in NMOSD-ON group, significantly different was found between onset and 1 m (*p* = 0.013), as well as between onset and 3 m (*p* = 0.038). As to BCVA in IDON group, no significant difference of the BCVA was found between each follow-up.

### Correlation of MD, PSD of VF and BCVA (Table 2)

**Table 2:** Correlation of MD, PSD and BCVA at different follow-up in NMOSD-ON and IDON patients

**Time**	**Final MD**		**Final PSD**		**Final BCVA**	
	**c–c**	***P***	**c–c**	***P***	**c–c**	***P***
**NMOSD-ON**						
**Onset**	0.202	0.602	-0.362	0.338	0.233	0.172
**1 m**	0.505	0.020*	0.580	0.005*	0.668	< 0.001*
**3 m**	0.806	< 0.001*	0.692	0.001*	0.859	< 0.001*
**IDON**						
**Onset**	0.276	0.172	0.145	0.480	0.086	0.456
**1 m**	0.597	< 0.001*	0.405	0.002*	0.167	0.127
**3 m**	0.862	< 0.001*	0.780	< 0.001*	0.452	< 0.001*

*NMOSD-ON* Neuromyelitis optica spectrum disorder- optic neuritis, *IDON* Idiopathic optic neuritis, *MD* Mean defect, *PSD* Pattern standard deviation, *BCVA* Best corrected visual acuity (log MAR), *c–c* Correlation-coefficient, *P P* value, *onset* At onset of optic neuritis, *1 m* At 1 month after onset, *3 m* At 3 months after onset, *Final MD/PSD/BCVA* The MD/PSD/BCVA at 6–12 months after onset

^*^P < 0.05

In Table 2, a positive correlation was found between the MD at 1 m and final (c–c = 0.505, *p* = 0.020 for NMOSD-ON; c–c = 0.597, *p* < 0.001 for IDON), as well as between 3 m and ≥ 6 m (c–c = 0.806, *p* < 0.001 for NMOSD-ON; c–c = 0.862, *p* < 0.001 for IDON). A positive correlation was found in PSD between 1 m and final (c–c = 0.580, *p* = 0.005 for NMOSD-ON; c–c = 0.405, *p* = 0.002 for IDON), as well as between 3 m and final (c–c = 0.692, *p* = 0.001 for NMOSD-ON; c–c = 0.780, *p* < 0.001 for IDON). A positive correlation was found in BCVA between 1 m and final only in NMOSD-ON group (c–c = 0.668, *p* < 0.001). And a positive correlation was found in BCVA between 3 m and final (c–c = 0.859, *p* < 0.001 for NMOSD-ON; c–c = 0.452, *p* < 0.001 for IDON). However, no correlation in visual function (MD, PSD and BCVA) was found between onset and final (*p* > 0.05).

### Recovery pattern of VF defect (Fig. 3)

**Fig. 3:**
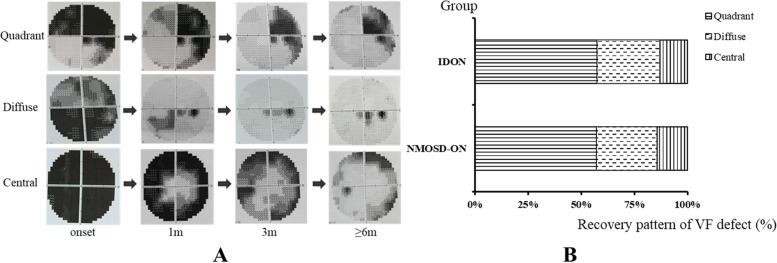
Recovery pattern of VF defect in NMOSD-ON and IDON. **A** The representative VF images of the three recovery patterns. **B** The distributions of the three recovery patterns in NMOSD-ON and IDON. The quadrant recovery pattern was found the most common in both groups and no significant difference was found between the two groups. NMOSD-ON: neuromyelitis optica spectrum disorder- optic neuritis, IDON: idiopathic optic neuritis, onset: at onset of optic neuritis, 1 m: at 1 month after onset, 3 m: at 3 months after onset, ≥ 6 m: at 6–12 months after onset

The main recovery patterns were found to be three forms, that is, central, quadrant and diffuse recovery. The representative VF images and distributions of the three recovery patterns were shown in Fig. 3. The quadrant recovery pattern was the most common pattern in two groups (57.1% in NMOSD-ON group versus 57.4% in IDON group). 28.6% in NMOSD group and 29.6% in IDON group were diffuse recovery. And 14.3% in the NMOSD group and 13.0% in IDON group were central recovery. But no significant difference in these recovery patterns was found between the two groups (*p* > 0.05).

### First episode NMOSD-ON and IDON (Table 3 and Fig. 4)

**Table 3:** Demographic features of first episode on NMOSD-ON and IDON

**Baseline features**	**NMOSD-ON (** ***n*** ** = 16)**	**IDON (** ***n*** ** = 52)**	***P***
**Age at onset (years)**	30.56 ± 16.99	32.79 ± 17.57	0.657
**Sex ratio F/M (%F)**	13/3 (81.3%)	25/27 (48.1%)	0.034*
**Affected eye by VF**			0.874
**Monocular**	9 (56.3%)	30 (57.7%)	
**Binocular**	7 (43.8%)	22 (42.3%)	
**Affected eye by BCVA**			0.214
**Monocular**	11 (68.8%)	43 (82.7%)	
**Binocular**	5 (31.2%)	9 (17.3%)	

*NMOSD-ON* Neuromyelitis optica spectrum disorder- optic neuritis, *IDON* Idiopathic optic neuritis, *P P* value

^*^P < 0.05

**Fig. 4:**
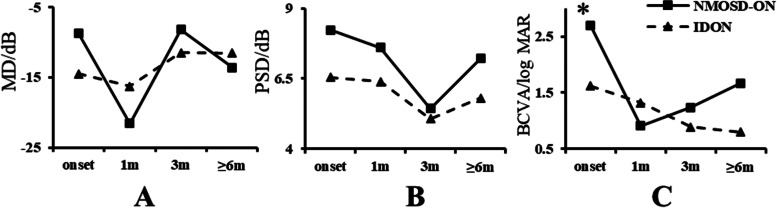
Evolvement of MD, PSD of VF and BCVA in first-ever NMOSD-ON and IDON. **A** The evolvement of MD in first-ever NMOSD-ON and IDON. At 1 m and ≥ 6 m, the MD in the NMOSD-ON group was lower than the IDON group. But at onset and 3 m, the MD in the NMOSD-ON group was higher than the IDON group. **B** The evolvement of PSD in first-ever NMOSD-ON and IDON. The PSD in NMOSD-ON group was higher than IDON group at onset and each follow-up of ON attack. **C** The evolvement of BCVA in first-ever NMOSD-ON and IDON. In the NMOSD-ON group, BCVA was only significantly worse than the IDON group only at onset. NMOSD-ON: neuromyelitis optica spectrum disorder- optic neuritis, IDON: idiopathic optic neuritis, MD: mean defect, PSD: pattern standard deviation, BCVA: best corrected visual acuity (log MAR), *P*: *P* value, *P < 0.05, onset: at onset of optic neuritis, 1 m: at 1 month after onset, 3 m: at 3 months after onset, ≥ 6 m: at 6–12 months after onset

The demographic data of first episode NMOSD-ON and IDON patients were summarized in Table 3. The female percentage of first episode NMOSD-ON was significantly higher than that of first episode IDON group (81.3% versus 47.2%, *p* = 0.034). No significant difference was found between the two groups as to age at onset and percentage of monocular or binocular involvement according to VF or BCVA change (*p* > 0.05).

We also monitored the changes of MD and PSD of VF and BCVA in first episode ON patients at onset and each follow-up showed in Fig. 4. No significant difference was found in MD and PSD of VF between the two groups at each time point (*p* > 0.05). The average BCVA in the NMOSD-ON group was worse than that in the IDON group at onset (log MAR, 2.69 ± 1.96 versus 1.62 ± 1.92, *p* = 0.028). No statistical difference was found in BCVA at 1 m, 3 m and ≥ 6 m between two groups (*p* > 0.05). The tendency of changes in the severity of VF defect and BCVA is consistent with the changes in overall samples between the two groups.

The VF defect patterns of first episode ON patients were analyzed. The proportion of eyes that failed the VF exam was higher in the NMOSD-ON group than that in the IDON group at onset (65.2% versus 35.1%, *p* = 0.011) and at ≥ 6 m (26.1% versus 5.4%, *p* = 0.014). Central scotoma was not found in the NMOSD-ON group, and only appeared in the IDON group. And the percentage of Arcuate and multifocal pattern were found more in the IDON group. But no significant difference could be found in these patterns above and other patterns between two groups (*p* > 0.05).

## Discussion

To the best of our knowledge, this is the first longitudinal prospective study of the VF characteristics of NMOSD-ON during acute attack with a follow-up period at least 6 months after onset. The VF defect of ON, pattern as well as severity, evolves during the process of acute attack until the inflammation finally stables. The results of VF might be influenced by the course, treatment regimen, experience of VF readers et al., among which course was the main factor. This study clarified the VF defect pattern, severity and recovery trend of two types of ON with different pathogenesis mechanism (astrocyte disease versus primary demyelination).

### Pattern of VF defect

VF not only reflects the severity of ON damage, but also reflects the localization of optic nerve damage by pattern. Most previous studies of VF defect pattern in acute phase of IDON reported that central scotoma was the dominant pattern at onset [12, 19, 20], but diffuse or nerve fiber bundle abnormality dominated 6 months after onset [10, 11], particularly when tested by Goldmann perimeter. Our study by static perimeter with 30–2 test program showed that diffuse abnormality at onset of VF defect in IDON group gradually evolved to nerve fiber bundle abnormality (18.9%), neurologic defect (13.9%) and central scotoma (8.9%) at ≥ 6 m. So, it was nerve fiber bundle abnormality rather than central scotoma was the dominant defect in the IDON group at ≥ 6 m. The difference might be due to the different devices that were used. Also, the pattern difference might also be influenced by the course of ON attack. A central scotoma mainly caused by the involvement of papillomacular bundles, may be covered by the diffuse involvement at onset. Therefore, if the VF of the enrolled patients were examined in early stage (within 8 days), the pattern of VF defect was diffuse abnormality. While the VF examined in later stage of ON (14–28 days or more), the pattern may be central scotoma.

Study about the pattern of VF in the acute phase of NMOSD-ON was extremely rare. It was reported that 76% of the 51 NMO-ON attacks had central scotoma of VF defect by Goldmann manual perimeter [12]. However, the exact course at which the VF tested was not mentioned. Our study by static perimeter showed that although diffuse pattern dominated most of the acute phase in NMOSD-ON group, the most common pattern gradually evolved to nerve fiber bundle abnormality (23.9%) at ≥ 6 m, rather than central scotoma. The percentage of neurologic abnormality of VF defect (quadrant, altitudinal, hemianopia and three quadrants pattern) in NMOSD-ON was higher than that of IDON. At 1 m, the altitudinal hemianopia in NMOSD-ON was found to be significantly higher than that of IDON. This finding was consistent with Nakajima et al.’s study [12], showing whenever visual acuity permitted, NMO patients showed much higher neurologic abnormality of VF defect, 10% for altitudinal, 6% for quadrant, 4% for three quadrants, 2% for hemianopia, and 2% for bitemporal hemianopia, while these patterns were extremely rare in MS-ON. However, in ONTT [4] study which contained mostly IDON, 14.9% of eyes showed altitudinal pattern at baseline. Therefore, the altitudinal pattern might not be characteristic of NMOSD-ON. Besides differences in neurologic abnormality pattern, NMOSD-ON also showed much less central scotoma of VF defect than that in IDON (15.8% in IDON group versus 0% in NMOSD-ON group), as early as at 1 m. This might indicate that the probability of papillomacular bundles involvement occurring alone in IDON, and its recovery might be earlier than other patterns defect in IDON. As to the mechanism of neurologic abnormality of VF defect, since NMOSD-ON is subject to damage the posterior part of orbital segment of optic nerve, and the nerve fiber bundles mainly run on the lateral side of it. So, whether this part is particularly prone to be attacked by AQP4-Ab due to the weakness of blood-optic nerve barrier remains to be investigated. As seen from the analyses above, the pattern of VF defect is helpful to diagnose and distinguish optic neuritis clinically.

### Recovery pattern of VF defect

This pattern of recovery might indicate which part of the optic nerve tends to recover first: from peripheral to central, vice versa, or quadrantal, and might indicate the pathological process involved during recovery. Our results showed that both types of ON mainly recovered quadrant-wise, without a trend of clockwise or counter-clockwise. But due to the small sample size, further investigation was needed to make a definite conclusion and the mechanism remained to be investigated.

### Severity and prognosis of VF

Numerous studies [10, 11, 21] showed that the visual function prognosis of NMOSD-ON was worse than that of IDON. The final MD of NMOSD-ON varied between -4.76 ~ -10.8 [10, 11, 21], while that of IDON varied between -4.6 ~ 7.9 [10, 11]. In this study, the severity of VF damage in NMOSD-ON was significantly more severe than that of IDON at all follow-ups, and there were significantly higher percentage of patients of NMOSD-ON (19.6%) still failed VF exam at ≥ 6 m supported that NMOSD-ON had a worse prognosis. However, there was no consensus as to the time point when the process of recovery terminated in ON (the end of acute phase), especially in NMOSD-ON. As to IDON, it was usually defined as 6 m ~ 9 m after onset [10, 11], while in NMOSD-ON, this time point varied in literature from 3 m ~ 8 m [10, 11, 21]. In this study, by detailed and regular longitudinal observation of BCVA, VF it was found that the recovery process of NMOSD-ON might terminated as early as 1 m after onset. The short period of recovery in NMOSD suggests that early treatment may be most effective.

The return of visual field function after acute optic neuritis was independent of the depth of the original defect (diffuse or localized VF defects), except for patients with the most severe defects [22]. This study also demonstrated that there was no correlation between severity of visual damage (BCVA, MD as well as PSD) at onset and that at the end of acute phase. However, a positive correlation detected between MD at 1 m and final MD was found in NMOSD-ON. A similar correlation was found for BCVA in NMOSD-ON. This was further support that the visual function of NMOSD-ON recovered as early as 1 m after onset and the visual function at this time point might be an indicator of the prognosis in NMOSD-ON. However, a correlation with final visual function of IDON was only found later (at 3 m), indicating a slower process in BCVA recovery.

### PSD of VF

There was little research about the PSD of VF in ON, which is an index for VF irregularity. Merle [10] found the PSD was significantly higher in MS than that in NMO among the first episode, while no significant difference was found in relapsed ON. This was contradictory to our study, which found PSD higher in NMOSD-ON than that in IDON at each follow-up. The difference in PSD of these two studies may be due to a difference in baseline severity of the disease. But the statistically significant difference of PSD was not found in the first episode ON patients. In this study, PSD reduced gradually during follow-ups in both groups. The tendency of PSD in the two groups showed that the irregularity of local VF was getting smaller with the recovery of ON. Throughout the course of the disease, the irregularity of local VF in NMOSD-ON patients was more obvious than that in IDON, suggesting the fact that NMOSD-ON may lead to more severe VF damage. A positive correlation was found between 1 m and final PSD, as well as between 3 m and ≥ 6 m. This might indicate the visual function at 1 m after onset is an indicator of the final prognosis in NMOSD-ON and IDON as the change of MD above.

### BCVA recovery

The BCVA recovery course of NMOSD-ON and IDON had seldom been reported before. Hiroki Masuda [9] reported that BCVA recovery from ON was poorer in NMOSD than that in MS and was negatively affected by pervious ON attack, but no longitudinal VF study was found. In this study during the follow-ups, there was an obvious different trend of BCVA and VF recovery between the two groups. In IDON, the recovery manifested a gradual and smooth trend. However, in NMOSD, the recovery manifested as a prompt recovery at 1 m then leveled off. And a positive correlation was found between at 1 m and final BCVA in NMOSD-ON group only. While a positive correlation was found between 3 m and final BCVA in IDON. This might indicate that the visual functions of 1 m in NMOSD-ON and 3 m in IDON were related to its prognosis.

### Visual function in first episode ON

Analyzing the VF defect in the first episode ON attack could exclude the influence from previous attacks. In this study, VF and BCVA damage in the first episode ON sub-group were more severe in NMOSD-ON than that of IDON, just as that in the mix group. Although only BCVA showed significant difference, it might be due to the sample size being not large enough. Our results were consistent with the other two studies. In Merle et al.’s study [10], for the eyes of first episode ON, ETDRS visual acuity, contrasts vision (Sloan chart 1.25%), MD in the NMO group were worse than that in MS-ON. However, no difference in visual acuity (Snellen) and retinal nerve fiber layer (RNFL)was found between them. In Fernandes et al.’s study [11], the number of eyes with normal VF (*p* < 0.001) and average VF MD (better than -3.0) after a single episode of ON (*p* < 0.001) was significantly less in NMO-ON than that in MS-ON. These results further supported that NMOSD-ON has more severe visual damage and poorer recovery than that in IDON. As to the pattern of VF in first episode ON, no significant difference could be found between the sub-groups in this study. However, central scotoma was only seen in IDON. This was consistent with previous studies indicating that central scotoma was the characteristic VF defect pattern in MS [12, 19]. The reason why papillomacular bundles were more vulnerable to damage in IDON was still unclear.

### Limitations

There were several limitations to our study. First, single center design might cause a certain degree of patient selection bias. Second, the sample size was not large enough. In our study, many VF subcategories had very small sample size, which disables the statistical comparison between the two groups. A larger sample size is needed to see if there were real differences. We believed that if the amount of patients with first episode was larger, more differences might be revealed between the two groups. Third, more indexes of visual function, e.g., color vision and contrast sensitivity, as well as structure index, e.g., RNFL and ganglion cell layer (GCL) analyses, should be observed. The correlation between these indexes should be analyzed to further understand the clinical characteristics of NMOSD-ON. Last, the data of another common type of ON, MOGAD related ON, was not included in this study. More prospective, multicenter, longitudinal cohort clinical study with larger sample size of patients of different types of ON has already been under investigation in our group.

## Conclusions

The NMOSD-ON patients tended to suffer more severe VF damage, VF irregularity and worse prognosis than that of IDON patients. Diffuse abnormalities and nerve fiber bundle abnormalities were the two most common types in both groups, while neurologic abnormality more common in NMOSD-ON and central scotoma more common in IDON.The visual functions of 1 m in NMOSD-ON and 3 m in IDON were related to its prognosis.

## Data Availability

The datasets used and analysed during the current study available from the corresponding author on reasonable request.
